# Development of Channelized K/V Band Dicke Microwave Radiometer Based on SDR

**DOI:** 10.3390/s26103059

**Published:** 2026-05-12

**Authors:** Zhenzhen Liang, Wei Guo, Caiyun Wang, Peng Liu, Shijie Yang

**Affiliations:** 1 University of Chinese Academy of Sciences, Beijing 100049, China; liangzhenzhen211@mails.ucas.ac.cn (Z.L.); yangshijie21@mails.ucas.ac.cn (S.Y.); 2 National Space Science Center, Chinese Academy of Sciences, Beijing 100190, China; wangcaiyun@mirslab.cn (C.W.); liupeng@mirslab.cn (P.L.)

**Keywords:** Dicke radiometer, software-defined radio (SDR), polyphase filter bank, channelized receiver

## Abstract

With the rapid development of software-defined radio (SDR) technology, a digital, software-reconfigurable, and flexible solution is provided for microwave radiometers, particularly suitable for atmospheric water vapor and oxygen detection with wideband, multi-channel requirements, significantly improving system efficiency. Meanwhile, digitization helps improve channel consistency and address nonlinearity issues, while the digital zero-balancing mechanism implemented through adaptive integration is more suitable for digital platforms. This paper proposes a digital Dicke-type radiometer system based on an SDR platform, using Xilinx RFSoC XCZU47DR (AMD, San Jose, CA, USA) as the core hardware to achieve single-chip integration of RF signal sampling, digital local oscillator generation, and signal processing. The system implements a 46-channel channelized receiver (23 channels each for K-band and V-band) on an FPGA using a polyphase filter bank. The prototype filters achieve 70 dB stopband attenuation and 0.5 dB passband ripple, with each polyphase branch requiring only 25 coefficients, significantly reducing hardware resource consumption. An adaptive integration method is proposed, where an adaptive switch controller dynamically adjusts the hot source injection time ratio by calculating the power difference between adjacent integration periods, enabling the Dicke zero-balancing mechanism to operate entirely in the digital domain. Furthermore, a complete hardware transfer model is established for three signal branches (antenna, hot source, and matched load), and full-chain calibration of all 46 channels is performed using a liquid nitrogen cold source, with calibration reliability verified through blackbody measurements. Experimental results demonstrate brightness temperature consistency better than 0.7 K, with a sensitivity of less than 0.15 K for the K-band and less than 0.21 K for the V-band at 1 s integration time.

## 1. Introduction

Microwave radiometers are high-sensitivity receivers designed to measure the natural thermal electromagnetic radiation of target scenes in the microwave frequency band. They have been widely applied in meteorological observation, environmental monitoring, remote sensing detection, and astronomical research [1]. The development of radiometer technology has been closely tied to advancements in modulation and balancing techniques. In 1946, Dicke pioneered the modulation-type radiometer architecture, in which the receiver periodically switches between the antenna signal and a reference load signal, thereby converting the influence of gain fluctuations into common-mode noise and effectively suppressing it. This classic design laid the foundation for modern radiometer technology [2]. In 1967, Goggins further advanced the zero-balancing technique by injecting controllable noise power into the transmission line through a variable attenuator, establishing a linear relationship between the radiometer output and the antenna temperature [3]. In 1974, Hardy et al. improved this method by utilizing semiconductor diodes to generate narrow-pulse noise signals and controlling the injected noise power by varying the pulse repetition frequency rather than pulse amplitude, substantially enhancing the system’s response speed, dynamic range, and long-term stability [4].

In recent years, extensive research has been conducted to improve radiometer system performance. Camps (2010) established a complete noise-wave analysis model based on S-parameters and temperature gradients, enabling post-processing correction of system errors through software algorithms [5]. Li et al. (2016) proposed a novel Dicke radiometer design that replaced the traditional active temperature control scheme with real-time detection of the reference load temperature, effectively simplifying the system structure and reducing power consumption [6]. Coto et al. (2019) derived an analytical expression for the nonlinear gain function of L-band radiometers, improving calibration accuracy through an accurate inverse transformation between the count domain and antenna temperature [7]. Lee and Popović (2023) developed a compact GaAs monolithic low-noise amplifier achieving 45.2 dB gain and 1.04 dB noise figure at 1.4 GHz [8]. Lee et al. (2025) further applied this technology to a 1.4 GHz Dicke radiometer for in-body temperature measurement, demonstrating the potential of radiometer technology in biomedical applications [9]. These efforts have primarily focused on analog front-end optimization and post-processing calibration algorithms.

**Figure 1:**
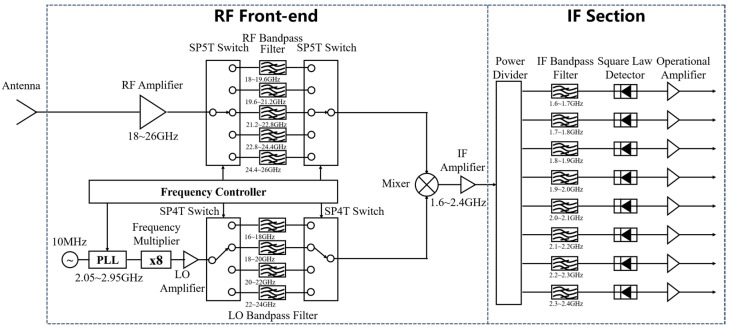
Block diagram of the conventional analog multi-channel radiometer architecture [10].

Accurate measurement of downward atmospheric radiative brightness temperature and inversion of atmospheric temperature and humidity profiles are of great significance for numerical weather prediction, climate research, and atmospheric environmental monitoring. The K-band (18–27 GHz) and V-band (50–75 GHz) are two core frequency bands for atmospheric sounding using microwave radiometers: the K-band radiometer performs remote sensing by utilizing the strong water vapor absorption line near 22.235 GHz and its wing channels, while the V-band radiometer is based on the complex absorption band structure of oxygen molecules near 60 GHz; both bands cover approximately 10 GHz wide strong absorption regions. To precisely establish the correspondence between absorption spectra and frequencies, it is necessary to synchronously acquire the entire 10 GHz bandwidth and divide it into multiple sub-bands for processing, which imposes demanding requirements on radiometer bandwidth and multi-channel observation capability. The traditional multi-channel analog radiometer architecture [10] faces severe challenges, as shown in Figure 1. First, conventional channelized receivers require an independent analog filter chain for each channel; as the number of channels increases, hardware complexity rises dramatically, making inter-channel consistency maintenance increasingly difficult. Second, the cumulative nonlinear effects of numerous analog components such as mixers, detectors, amplifiers, and filters degrade system linearity and measurement accuracy. Furthermore, the complex analog architecture results in large system weight and volume, which is unfavorable for the miniaturization and lightweight design required by airborne and spaceborne applications. The digital Dicke radiometer can effectively solve the problems of inter-channel imbalance, large weight, and bulk volume.

When implementing digital channelization on FPGA, conventional schemes consume a substantial number of multipliers due to high filter orders, and the combination of high clock frequencies and large resource requirements often leads to timing closure failures [11]. Second, the zero-balancing mechanism in traditional Dicke radiometers relies on analog pulse-noise injection and hardware-based feedback loops. This approach is inherently tied to analog components such as variable attenuators and directional couplers, making it difficult to directly migrate to a fully digital architecture [12]. How to achieve efficient and stable zero-balancing entirely in the digital domain remains a key challenge for the digitalization of Dicke radiometer systems [13].

Polyphase filtering is one of the core techniques in digital channelized receivers. In 1974, Bellanger et al. first proposed the cascade combination of polyphase networks and the discrete Fourier transform, establishing the theoretical foundation for polyphase filter bank channelization [14]. By moving the decimation operation ahead of data processing, this technique effectively reduces the data processing rate and hardware resource consumption, providing a practical approach for wideband receiver implementation [15]. Currently, digital channelization based on polyphase filter banks has been widely applied in communications, radar, and electronic reconnaissance systems [16]. Meanwhile, the emergence of Radio Frequency System-on-Chip (RFSoC) devices, which integrate high-speed data converters and programmable logic resources on a single chip, has provided a new hardware foundation for further integration of digital channelized receivers.

This paper applies polyphase filter bank channelization to Dicke radiometers and proposes a compatible digital zero-balancing method, constructing a fully digital system after down-converted to the SDR sampling range. This paper adopts the Xilinx Zynq UltraScale+ RFSoC XCZU47DR as the hardware platform. The XCZU47DR (AMD, San Jose, CA, USA) is a third-generation RFSoC device that integrates 8-channel 14-bit analog-to-digital converters (ADCs) with a maximum sampling rate of 5 GSPS, 8-channel 14-bit digital-to-analog converters (DACs) with a maximum sampling rate of 9.8 GSPS, a quad-core ARM Cortex-A53 processor (ARM, Cambridge, UK), and large-scale FPGA programmable logic resources on a single chip [17]. This high level of integration enables the consolidation of RF signal sampling, digital channelization processing, local oscillator generation, and system control onto a single device, substantially reducing system size and inter-module interconnect complexity compared to conventional multi-board architectures. Based on this platform, this paper proposes a digital Dicke-type radiometer system covering both K-band (21.3–31.7 GHz) and V-band (50.8–61.2 GHz) with a total of 46 channels. The main contributions of this work are as follows:

(1) A polyphase filter bank channelized receiver is implemented on the FPGA of the XCZU47DR, dividing the wideband signal into 46 channels with 23 channels per band. By decomposing high-order prototype filters into parallel sub-filter structures, each polyphase branch requires only 25 coefficients while achieving 70 dB stopband attenuation and 0.5 dB passband ripple, significantly reducing FPGA resource consumption and resolving timing closure difficulties inherent in conventional channelization schemes.

(2) An adaptive integration method is proposed to achieve digital zero-balancing on the SDR platform. The adaptive switch controller calculates the power difference between adjacent integration periods and dynamically adjusts the hot source injection time ratio for the next cycle, enabling the Dicke zero-balancing mechanism to operate entirely in the digital domain without relying on analog noise injection hardware.

(3) A complete hardware transfer model is established for all three signal branches (antenna, hot source, and matched load), accounting for transmission losses, reflection coefficients, and physical temperature contributions of each RF front-end component. Full-chain calibration is performed on all 46 channels using a liquid nitrogen cold source, and calibration reliability is verified through blackbody measurements, demonstrating brightness temperature consistency better than 0.7 K, with a sensitivity of less than 0.15 K for the K-band and less than 0.21 K for the V-band at 1 s integration time.

Previously, SDR-based radiometers mostly operated in narrowband, low-channel-count configurations. Farhad et al. [18] implemented an L-band radiometer covering only 27 MHz of protected spectrum with a 30 MHz sampling rate in a single-receive-channel architecture; Al Mahmud and Kurum [19] achieved 400 MHz spectrum monitoring in S-band through segmented scanning, also with 30 MHz sampling rate, but still processed only a single channel at a time. In contrast, the proposed system leverages the 5 GSPS ADC of RFSoC XCZU47DR to achieve approximately 10.4 GHz of instantaneous RF bandwidth per band, and partitions the wideband signal into 46 channels (23 per band) in real time via polyphase filter banks on FPGA. The proposed system exceeds existing SDR radiometer architectures by several orders of magnitude in both instantaneous bandwidth and number of simultaneously operating channels.

Regarding the zero-balancing mechanism, existing SDR radiometers have not achieved true closed-loop Dicke zero-balancing. Farhad et al. [18] employed fixed 250 ms sequential port switching, relying entirely on post-measurement calibration correction; Al Mahmud and Kurum [19] drove SP3T relay switching at preset intervals with predetermined duty cycles, where measurement results did not participate in feedback updating of control parameters. In comparison, the adaptive integration method proposed in this paper fully reproduces the fundamental principle of Dicke zero-balancing in the digital domain: after each integration period, the system computes the power difference between the antenna branch and matched load branch, multiplies this difference by an adaptive coefficient k to determine the adjustment amount for the heat source injection time in the next period, and applies the updated switching time ratio to the SPDT switch through the GPIO interface. All computation and control decisions are completed within the FPGA without any analog feedback components, which represents the first fully digitalized closed-loop Dicke zero-balancing mechanism implemented on an SDR platform.

The remainder of this paper is organized as follows. Section 2 presents the development of the dual-band radiometer system, comprising the antenna unit, the analog unit, and the digital unit. The digital unit is detailed with the polyphase filter bank channelization (Section 2.3.3) and the adaptive integration method (Section 2.3.5). Section 3 describes the calibration methodology, including the radiative transfer model establishment (Section 3.1) and the calibration procedure (Section 3.2). Section 4 shows the experimental results, covering the calibration performance analysis (Section 4.1) and a comparison with existing radiometer systems (Section 4.2). Section 5 discusses the key findings, and Section 6 concludes the paper.

## 2. System

**Figure 2:**
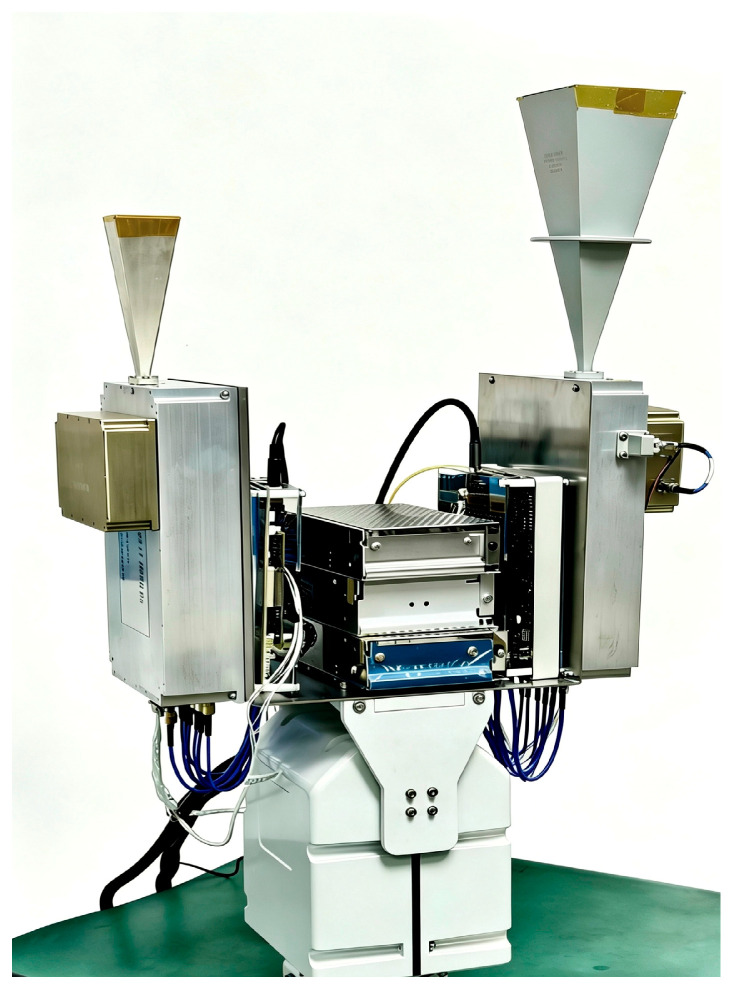
Photograph of the radiometer.

The radiometer developed in this paper is shown in Figure 2.

**Figure 3:**
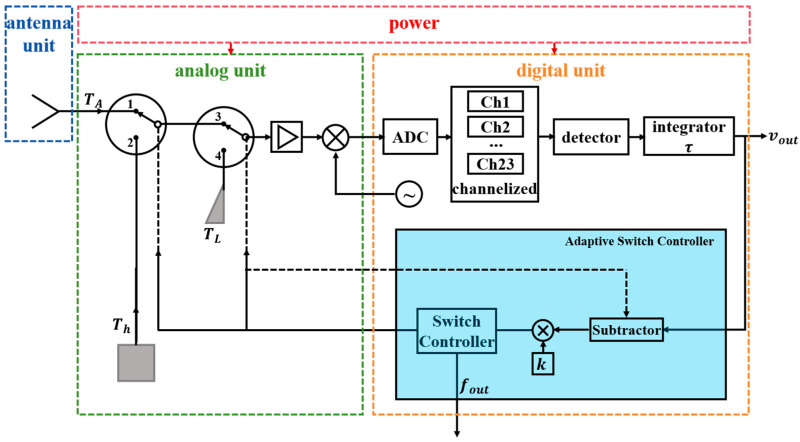
Brief block diagram of the system.

The Dicke radiometer consists of four parts: the antenna unit; the analog unit; the digital unit; and the power, as shown in Figure 3. The analog unit switches signal sources through two single-pole double-throw (SPDT) switches: when the switches connect to ports 1 and 3, the antenna branch is connected; when connected to ports 2 and 3, the hot source branch is connected; when connected to port 4, the matched load branch is connected. The total integration time of the antenna branch and the high-temperature source branch equals that of the matched load branch.

The digital unit is implemented based on a software-defined radio (SDR) architecture. The analog unit first down-converts the signal into the SDR sampling bandwidth, with subsequent processing completed by the digital unit. The digital unit employs a channelized receiver to divide the wideband signal into 23 channels, improving the modulation scheme to make the zero-balancing process suitable for digital platforms. After independent detection and integration in each channel, the signals are sent to an adaptive switch controller to achieve digital zero-balancing. This controller comprises a subtractor, a multiplier, and a switch controller: the subtractor calculates the power difference between adjacent integration periods when the switches connect to ports 3 and 4; the multiplier multiplies this difference by an adjustment coefficient k to obtain the time ratio for connecting ports 1 and 2 in the next cycle. The specific calculation method for coefficient k will be detailed in the Section 2.3.5. Finally, the switch controller drives the two switches in the analog unit to complete the adaptive digital zero-balancing.

The channel frequency allocations are listed in Table A1, with each channel having a bandwidth of 288 MHz or 400 MHz. The key technical specifications of the developed K/V dual-band SDR-based radiometer are summarized in Table 1.

### 2.1. Antenna

**Figure 4:**
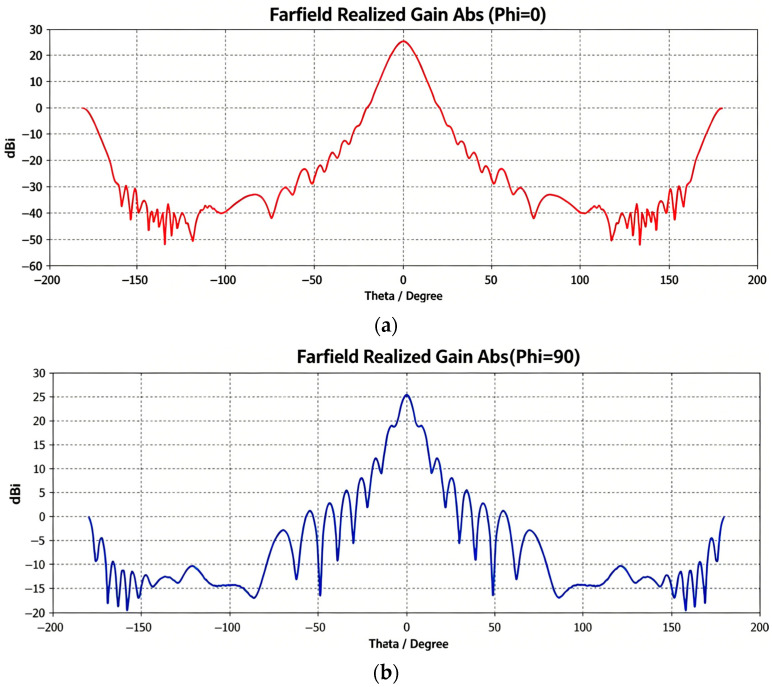
Principal plane radiation patterns of the K-band antenna at 25 GHz: (**a**) Phi = 0; (**b**) Phi = 90.

**Figure 5:**
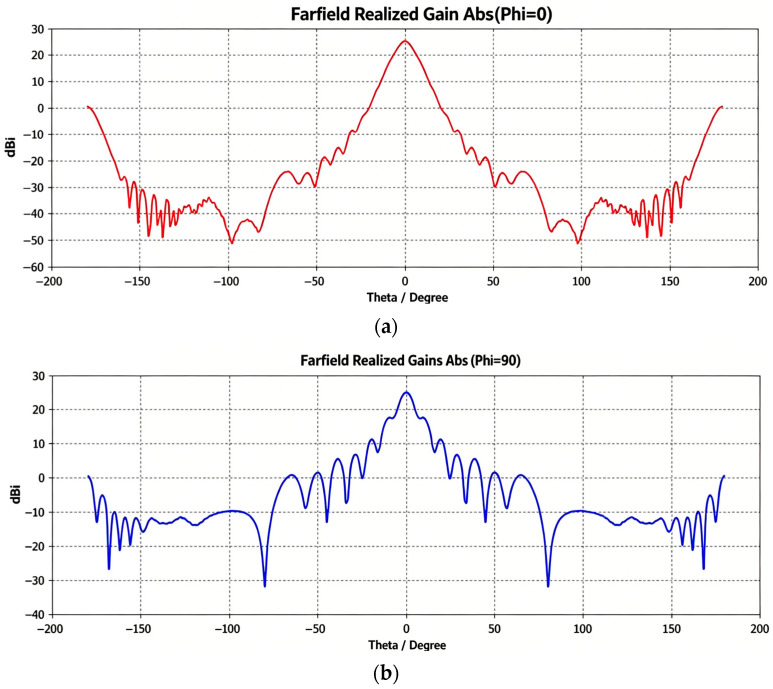
Principal plane radiation patterns of the V-band antenna at 56 GHz: (**a**) Phi = 0; (**b**) Phi = 90.

The antenna parameters are listed in Table 2. Both the K-band and V-band antennas exhibit a gain of 25 dBi and a VSWR (Voltage Standing Wave Ratio) better than 1.25. The principal plane radiation patterns at Phi = 0° and Phi = 90° for K-band and V-band antennas are shown in Figure 4 and Figure 5, respectively.

### 2.2. Radiometer Receiver Analog Unit

**Figure 6:**
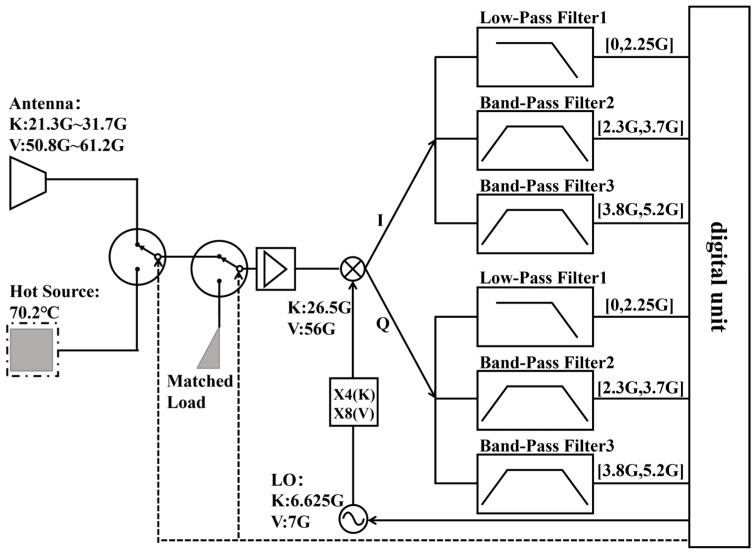
Schematic diagram of receiver analog unit.

As shown in Figure 6, the antenna port, matched load port, and hot source port are switched via two single-pole double-throw (SPDT) switches. The K-band antenna covers 21.3–31.7 GHz, and the V-band antenna covers 50.8–61.2 GHz; the hot source is placed in a temperature-controlled chamber. The selected signal is amplified by a low-noise amplifier (LNA) and then enters a harmonic mixer to complete the first down-conversion. The local oscillator (LO) chain employs a frequency multiplication scheme: the K-band fundamental frequency of 6.625 GHz is quadrupled to generate a 26.5 GHz LO; the V-band fundamental frequency of 7 GHz is multiplied by eight to generate a 56 GHz LO. The mixer output produces quadrature I/Q intermediate frequency (IF) signals, which respectively pass through low-pass filter 1: DC–2.25 GHz, band-pass filter 2: 2.30–3.70 GHz, and band-pass filter 3: 3.80–5.20 GHz. These six baseband analog signals are sent to the digital unit for subsequent digital signal processing.

### 2.3. Radiometer Receiver Digital Unit

**Figure 7:**
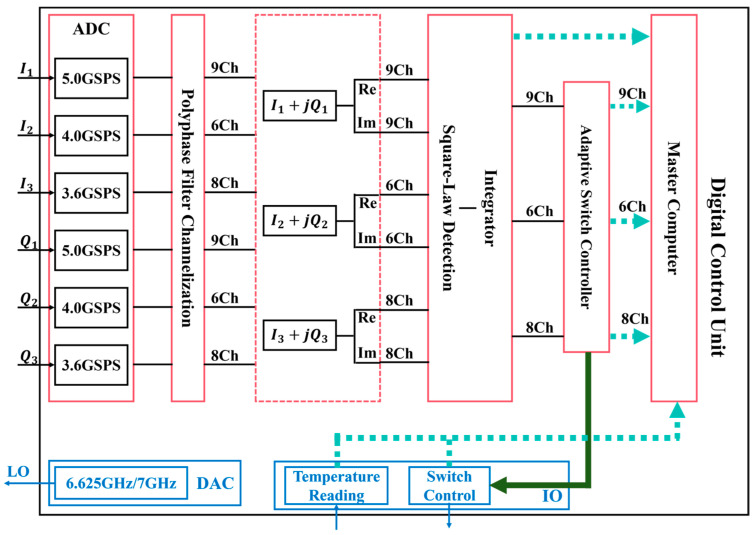
Digital unit of the radiometer receiver.

The digital unit comprises an ADC module, a polyphase filter channelization module, an I/Q combination module, a square-law detection module, an integrator module, an adaptive switch controller, and a Master computer, as illustrated in Figure 7. The six-channel signals (three I and three Q) output from the analog front-end are sampled by ADCs, respectively, and then channelized by a polyphase filter bank into three sub-channel groups of 9, 6, and 8 paths. After I + jQ recombination, the final complex signals of 23 channels are obtained. The signals subsequently undergo square-law detection and integration. The adaptive switching control module calculates the switching timing relationship of the feedback loop to drive the analog front-end switches for digital zero-balancing. Finally, the integration results and switch status information are uploaded to the host computer for subsequent data processing and analysis.

Additionally, the digital unit realizes local oscillator output for the analog unit through the RF-DAC and implements temperature reading and switch control functions for the analog unit via the GPIO interface.

#### 2.3.1. Clock System

In this work, the TICS Pro tool from Texas Instruments (Dallas, TX, USA) is employed to configure the LMK04828 clock chip, which generates the operating clocks for the ADC and DAC. Subsequently, phase-locked loops (PLLs) are utilized to derive the various clocks required for digital signal processing.

PLL Configuration and VCO Locking: The system adopts an external 19.2 MHz VCXO as the reference input (OSCin), which is multiplied and locked to the internal VCO1 through the PLL. For PLL2, the N-divider is set to 125 and the R-divider to 4, yielding a phase detector frequency of 4.8 MHz with a charge pump current of 3200 μA. This configuration produces a VCO1 output frequency of 3000 MHz. This VCO frequency serves as the global clock source, and the required clocks for each branch are generated through the post-stage divider network. The 3000 MHz clock from VCO1 is divided by the Clock Divider, adjusted by Digital Delay and Analog Delay, and then routed through Clock Output Select to choose the output type. Finally, it is distributed to each ADC and DAC channel, with all output channels adopting the LVDS electrical standard [20].

#### 2.3.2. ADC and DAC Modules

The analog signals $I_{1}$ and $Q_{1}$ (hereinafter referred to as Channel I) have a frequency range of DC–2.2 GHz, with a sampling rate of 5.0 GHz. The ADC module clock is 500 MHz, with 10 signal samples processed in parallel per clock cycle. This configuration satisfies the oversampling condition, with the signal residing in the first Nyquist zone at DC–2.25 GHz. The analog signals $I_{2}$ and $Q_{2}$ (hereinafter referred to as Channel II) have a frequency range of 2.3–3.7 GHz, with a sampling rate of 4.0 GHz. The ADC module clock is 500 MHz, with 8 signal samples processed in parallel per clock cycle. This configuration results in undersampling, with the signal appearing in the second Nyquist zone. The corresponding frequency in the first Nyquist zone is 1.7–0.3 GHz. Note that spectral inversion occurs in this case. The analog signals $I_{3}$ and $Q_{3}$ (hereinafter referred to as Channel III) have a frequency range of 3.8–5.2 GHz, with a sampling rate of 3.6 GHz. The ADC module clock is 360 MHz, with 10 signal samples processed in parallel per clock cycle. This configuration also results in undersampling, with the signal appearing in the third Nyquist zone. The corresponding frequency in the first Nyquist zone is 0.2–1.6 GHz. The first and third Nyquist zones have consistent spectral orientation, so no spectral inversion occurs. The summary is presented in Table 3.

**Figure 8:**
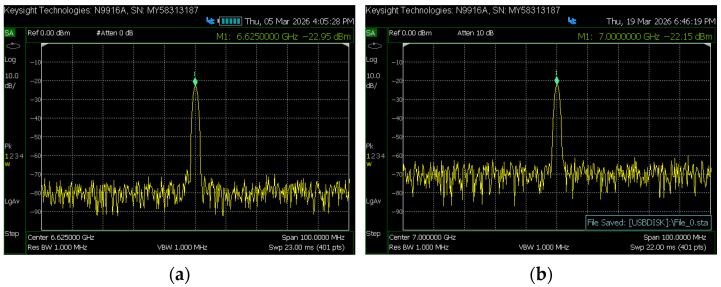
Digital local oscillator spectrum: (**a**) K-band local oscillator spectrum; (**b**) V-band local oscillator spectrum.

The DAC is utilized for the local oscillator (LO) output, with a sampling rate of 5.0 GSPS. For the K-band, the LO frequency is 6.625 GHz at a power level of −22.95 dBm, with spurious suppression of 47 dBm and no significant spurious components, as shown in Figure 8a. This 6.625 GHz signal is quadrupled in the analog unit to generate a 26.5 GHz LO, which drives the K-band mixer. For the V-band, the LO frequency is 7 GHz at a power level of −22.15 dBm, with spurious suppression of 43 dBm and no significant spurious components, as shown in Figure 8b. This 7 GHz signal is multiplied by eight in the analog unit to generate a 56 GHz LO, which drives the V-band mixer.

#### 2.3.3. Polyphase Filter Channelization

**Figure 9:**
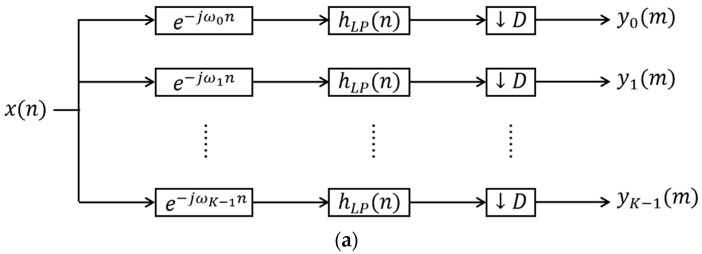

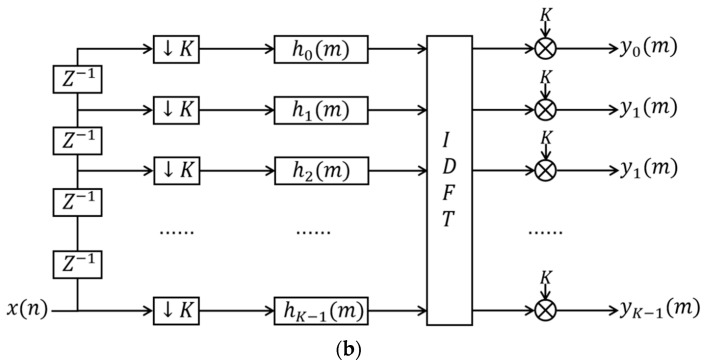
Channelized receiver scheme: (**a**) traditional channelized receiver scheme; (**b**) polyphase filter channelized digital receiver scheme.

In conventional channelization schemes implemented on an FPGA, the filter stage consumes a substantial number of multipliers, as shown in Figure 9a. Moreover, when stringent filter requirements necessitate a high filter order, combined with elevated clock frequencies, the development board resources become severely constrained, potentially leading to timing closure failures during implementation. Therefore, polyphase filters are adopted for the channelized receiver, as shown in Figure 9b, which effectively reduces resource utilization and resolves issues of insufficient resources and timing violations.

The ADC-output signals first undergo serial-to-parallel conversion to realize polyphase signal tapping: specifically, $I_{1}$ and $Q_{1}$ are converted into 10 parallel channels under the 500 MHz clock; $I_{2}$ and $Q_{2}$ are converted into 8 parallel channels under the 500 MHz clock; $I_{3}$ and $Q_{3}$ are converted into 10 parallel channels under the 360 MHz clock.

The polyphase-tapped signals are subsequently fed into the corresponding polyphase filters, which are obtained through polyphase decomposition of the prototype filters. The parameters of the prototype filters corresponding to the three wideband signals are listed in Table 4. All three prototype filters achieve a stopband attenuation of 70 dB, a passband ripple of 0.5 dB, and a rectangular ratio of 0.8. The number of coefficients for the three prototype filters are 250, 200, and 250, respectively. The polyphase decomposition channel numbers for the three prototype filters are 10, 8, and 10, respectively; consequently, each polyphase filter branch contains 25 coefficients.

**Figure 10:**
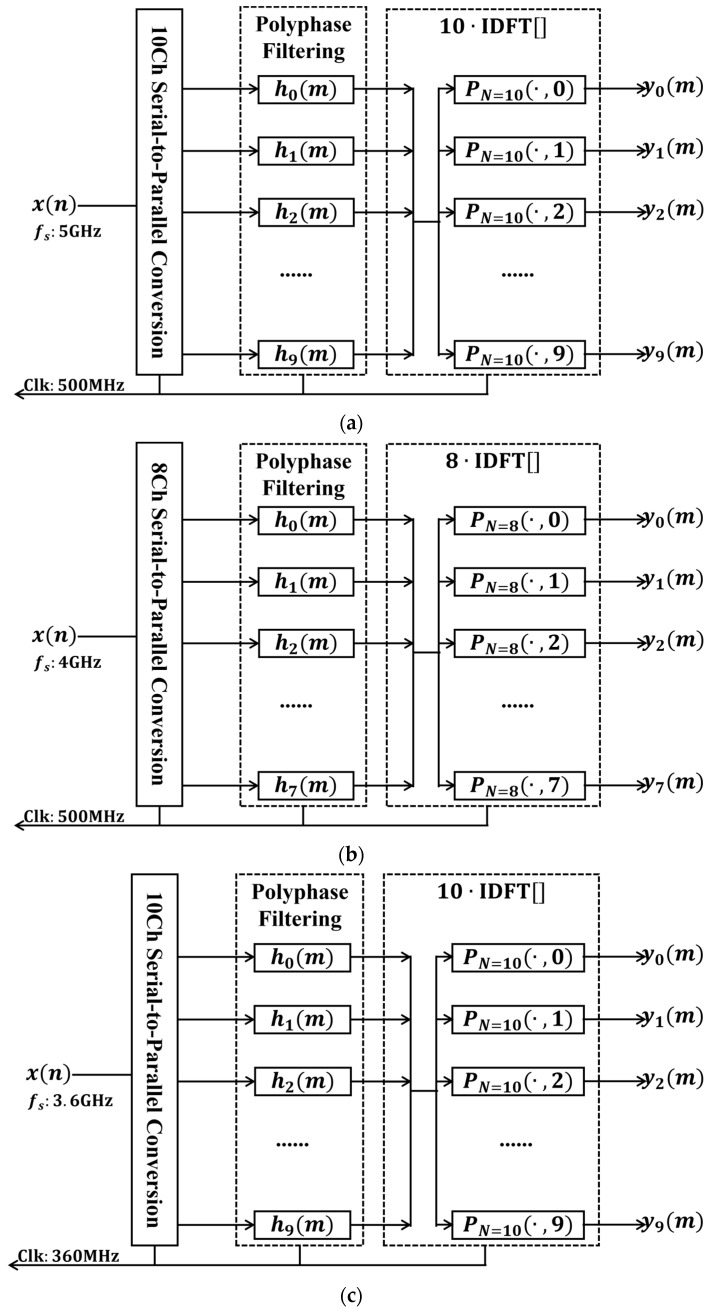
Multiphase filtering channelized process: (**a**) Channel I; (**b**) Channel II; (**c**) Channel III.

The signals processed by the polyphase decomposition filters are subsequently sent to the IDFT stage. Since direct IDFT computation requires a relatively large number of multipliers, this paper adopts multiple filters to replace the IDFT calculation. The computational equivalence between these two approaches is maintained, while the filter-based implementation offers greater operational convenience and resource efficiency. The channelization processes for the three wideband signals are summarized in Figure 10. Here, $P_{N=10}\;$ and $P_{N=8}\;$ are both complex numbers; therefore, $y_{k}\left(m\right)$ is complex for all cases. After performing polyphase filter channelization separately on the I-channel and Q-channel signals, further computation is required to obtain the true channelized signals. The I-channel signal after polyphase filter channelization is $Re[y_{k\_I}\left(m\right)]+jIm[y_{k\_I}\left(m\right)]$, and the Q-channel signal after polyphase filter channelization is $Re[y_{k\_Q}\left(m\right)]+jIm[y_{k\_Q}\left(m\right)]$. The combined result has a real part of $Re[y_{k\_I}\left(m\right)]-Im[y_{k\_Q}\left(m\right)]\;$ and an imaginary part of $Im[y_{k\_I}\left(m\right)]+Re[y_{k\_Q}\left(m\right)]$.

**Figure 11:**
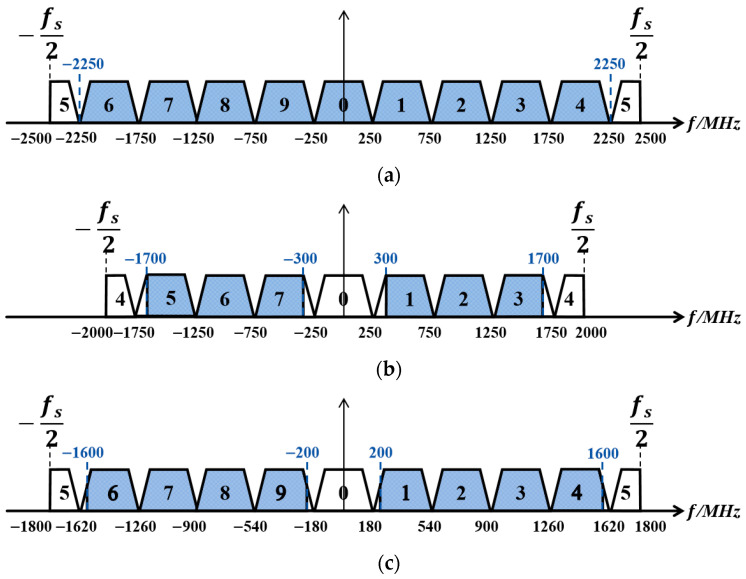
Multiphase filtering channel numbering: (**a**) Channel I; (**b**) Channel II; (**c**) Channel III.

The first complex wideband signal spans [−2.25 GHz, 2.25 GHz]; therefore, the fifth channel among its corresponding 10 channels contains no signal. When combining the I-channel and Q-channel signals, the fifth channel does not participate in the computation. Thus, the first complex wideband signal effectively yields 9 valid output channels, as shown in Figure 11a. The second complex wideband signal spans [−1.7 GHz, −0.3 GHz] and [0.3 GHz, 1.7 GHz]; therefore, the 0th and 4th channels among its corresponding 8 channels contain no signal. When combining the I-channel and Q-channel signals, the 0th and 4th channels do not participate in the computation. Thus, the second complex wideband signal effectively yields 6 valid output channels, as shown in Figure 11b. The third complex wideband signal spans [−1.6 GHz, −0.2 GHz] and [0.2 GHz, 1.6 GHz]; therefore, the 0th and 5th channels among its corresponding 10 channels contain no signal. When combining the I-channel and Q-channel signals, the 0th and 5th channels do not participate in the computation. Thus, the third complex wideband signal effectively yields 8 valid output channels, as shown in Figure 11c.

#### 2.3.4. Square-Law Detection and Integrator

**Figure 12:**
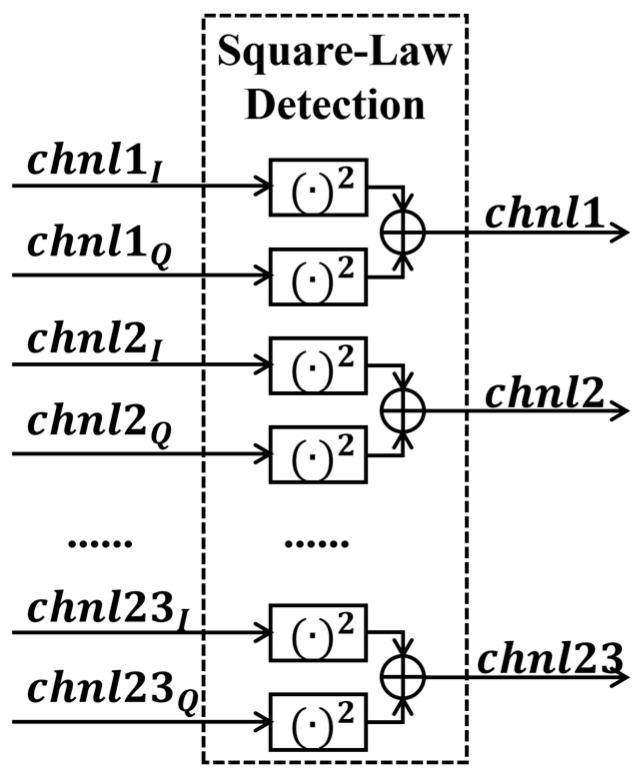
Square-law detection and integrator flowchart.

As shown in Figure 12, the real and imaginary parts of the 23-channel signals are both fed into the square-law detection module to compute the power of each channel. The detected power signals are then sent to the integrator module with a minimum integration time of 10 ms.

#### 2.3.5. Adaptive Switch Controller

**Figure 13:**
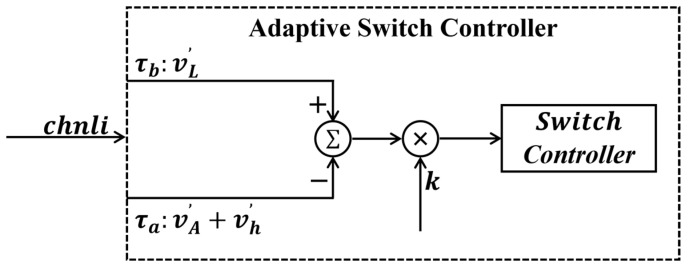
Adaptive switch controller flowchart.

Taking one channel as an example, the structure of the adaptive switch controller is illustrated in Figure 13. The integrated signal power is defined as follows: when switch 3 is turned on, the integrated power is denoted as $\left(v_{A}^{\prime }+v_{h}^{\prime }\right)$; when switch 4 is turned on, the integrated power is denoted as $v_{L}^{\prime }$. The power values of these two adjacent integration periods are subtracted. If the difference is greater than 0, the injection duration of the hot source needs to be increased in the next integration period, and vice versa.

**Figure 14:**
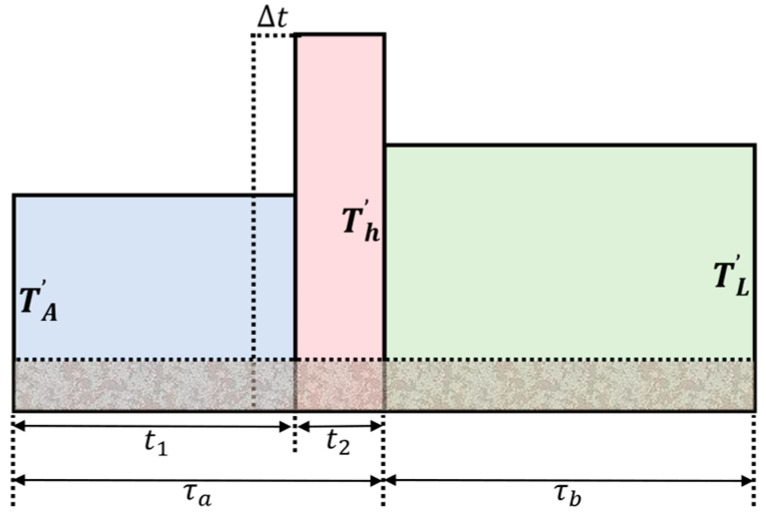
Schematic diagram of pulse injection power versus integration time.

This adjustment amount is denoted as ∆t, as shown in Figure 14. The gray shaded area in the figure represents the system noise floor, which remains consistent during $\tau_{a}$ and $\tau_{b}$, and thus can be canceled out. The key to achieving a zero balance using the adaptive integration method is to make the power values during periods $\tau_{a}$ and $\tau_{b}$ equal.

The power value that needs to be compensated in the next integration period is

(1) $$\left({T^{\prime }}_{h}-{T^{\prime }}_{A}\right)\cdot ∆t=v_{h}^{\prime }+v_{A}^{\prime }-v_{L}^{\prime }$$

where ${T^{\prime }}_{h}=v_{h}^{\prime }/t_{2}$, ${T^{\prime }}_{A}=v_{A}^{\prime }/t_{1}$, with $t_{1}$ and $t_{2}\;$ being the integration times of the switch in the antenna branch and the hot source branch, respectively, and $v_{h}^{\prime }$ and $v_{A}^{\prime }$ being the integrated values of the switch in the antenna branch and the hot source branch, respectively. Therefore, the multiplier coefficient k in Figure 13 is

(2) $$k=\frac{1}{{T^{\prime }}_{h}-{T^{\prime }}_{A}}$$

After passing through the multiplier, the hot source injection time that needs to be adjusted for the next integration period can be obtained. The time adjustment for the next period is

(3) $$\hat{t_{1}}=t_{1}-∆t$$

(4) $$\hat{t_{2}}=t_{2}+∆t$$

The adjusted switch control time takes effect in the next integration period, and the high/low levels are output through the IO port of the SDR development board to control the switches of the analog unit.

## 3. Calibration

Calibration consists of two steps: system self-calibration and cold source calibration. System self-calibration is performed to eliminate measurement errors caused by differences in transmission characteristics of electronic devices among the three branches during radiative transfer. Cold source calibration is used to determine and correct the actual input brightness temperature of the hot source, as the temperature at the hot source port is not completely equivalent to that of the constant temperature box, and its matching degree is lower than that of the matched load; therefore, determination and correction are required using the cold source. Finally, the calibration results are verified using a blackbody.

### 3.1. Hardware Transfer Model

**Figure 15:**
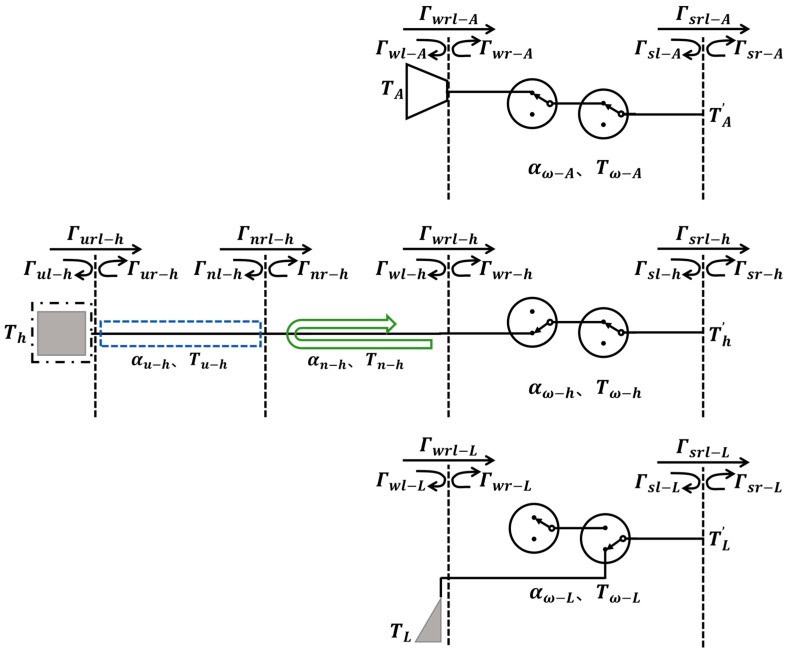
RF front-end transmission model of each branch of the radiometer.

The hardware transfer model of this radiometer is illustrated in Figure 15. In the antenna branch, $T_{A}$ denotes the input brightness temperature, $T_{\omega -A}$ is the physical temperature of the switch; at the ω reference plane, $\Gamma_{\omega l-A}$ and $\Gamma_{wr-A}$ are the power reflection coefficients looking from the left and right sides, respectively, $\Gamma_{\omega rl-A}$ is the power transmission coefficient.

In the hot source branch, $T_{h}$ is the temperature of the hot source module, $T_{u-h}$ is the physical temperature of the cable, $T_{n-h}$ is the physical temperature of the waveguide-to-coax adapter, and $T_{w-h}$ is the physical temperature of the switch; at the u reference plane, $\Gamma_{ul-h}$ and $\Gamma_{ur-h}$ are the power reflection coefficients looking from the left and right sides, respectively, $\Gamma_{url-h}$ is the power transmission coefficient; at the n reference plane, $\Gamma_{nl-h}$ and $\Gamma_{nr-h}$ are the power reflection coefficients looking from the left and right sides, respectively, $\Gamma_{nrl-h}$ is the power transmission coefficient; at the ω reference plane, $\Gamma_{wl-h}$ and $\Gamma_{wr-h}$ are the power reflection coefficients looking from the left and right sides, respectively, $\Gamma_{\omega rl-h}$ is the power transmission coefficient; and $\alpha_{u-h}$, $\alpha_{n-h}$, and $\alpha_{\omega -h}$ are the power transmission coefficients of the cable, waveguide-to-coax adapter, and switch, respectively.

In the matched load branch, $T_{L}$ is the physical temperature of the matched load; $T_{\omega -L}$ is the physical temperature of the switch; at the ω reference plane, $\Gamma_{wl-L}$ and $\Gamma_{wr-L}$ are the power reflection coefficients looking from the left and right sides, respectively, $\Gamma_{\omega rl-L}$ is the power transmission coefficient; and $\alpha_{\omega -L}$ is the power transmission coefficient of the switch.

The s reference planes of the three branches are the same point, therefore $\Gamma_{sl-A}=\Gamma_{sl-h}=\Gamma_{sl-L}$, $\Gamma_{sr-A}=\Gamma_{sr-h}=\Gamma_{sr-L}$, $\Gamma_{srl-A}=\Gamma_{srl-h}=\Gamma_{srl-L}$. At the s reference plane, $\Gamma_{sl-A}$ and $\Gamma_{sr-A}$ are the power reflection coefficients looking from the left and right sides, respectively, $\Gamma_{srl-A}$ is the power transmission coefficient; and $\alpha_{\omega -A}$ is the power transmission coefficient of the switch.

For the antenna branch, the antenna interface is a waveguide port connected to the antenna port of the receiver analog unit. To analyze the input brightness temperature ${T_{A}}^{\prime }$ at the receiver, the output ${T_{A}}^{\prime }$ from the RF front-end consists of three components: the first part is the direct output of the input brightness temperature $T_{A}$ after attenuation through the RF front-end; the second part is the contribution to ${T_{A}}^{\prime }$ from the radiated brightness temperature of each component in the RF front-end, directly transmitted backward; and the third part is the contribution to ${T_{A}}^{\prime }$ from the radiated brightness temperature of each component in the RF front-end reflected at interface w and then transmitted backward. The analysis yields

(5) $$T_{A}^{\prime }=\left({aa}_{A}\cdot T_{A}+{bb}_{A}\cdot T_{\omega -A}\right)\left(1-\Gamma_{sl-A}\right)$$

where

(6) $$\left\{\begin{array}{c} {aa}_{A}=\left(1-\Gamma_{\omega l-A}\right)\cdot \alpha_{\omega -A}\\ {bb}_{A}=\left(1-\alpha_{\omega -A}\right)+\left(1-\alpha_{\omega -A}\right)\cdot \Gamma_{wr-A}\cdot \alpha_{\omega -A} \end{array}\right.$$

For the hot source branch, the hot source module first passes through a cable, which is connected to a waveguide-to-coax adapter. The waveguide-to-coax adapter is then connected to the hot source interface of the receiver analog unit. Analyzing the brightness temperature $T_{h}^{\prime }$ after the isolator following the receiver switch, it consists of 7 components: the direct output of the hot source input brightness temperature after attenuation by the RF front-end; the output of the hot source input brightness temperature reflected by the w-reference plane through the cable and waveguide-to-coax adapter, then reflected by the n-reference plane and entering the RF front-end for attenuation; the direct output of the cable’s radiated brightness temperature after attenuation by the RF front-end; the direct output of the waveguide-to-coax adapter’s radiated brightness temperature after attenuation by the RF front-end; the output of the waveguide-to-coax adapter’s radiated brightness temperature reflected by the n-reference plane and entering the RF front-end for attenuation; the contribution of the brightness temperature of each component in the RF front-end directly transmitted backward; the contribution of the radiated brightness temperature of each component in the RF front-end reflected by the w-interface and then transmitted backward. The analysis yields

(7) $$T_{h}^{\prime }=\left({aa}_{h}\cdot T_{h}+{BB}_{h}\cdot T_{u-h}+{dd}_{h}\cdot T_{\omega -h}\right)\left(1-\Gamma_{sl-A}\right)$$

where

(8) $$\left\{\begin{array}{c} {aa}_{h}=\alpha_{u-h}\cdot \left(1-\Gamma_{nl-h}\right)\cdot \alpha_{n-h}\cdot \left(1-\Gamma_{wl-h}\right)\cdot \alpha_{\omega -h}+\alpha_{u-h}\cdot \left(1-\Gamma_{nl-h}\right)\cdot \\ \alpha_{n-h}\cdot \Gamma_{wl-h}\cdot \alpha_{n-h}\cdot \Gamma_{nr-h}\cdot \alpha_{n-h}\cdot \left(1-\Gamma_{wl-h}\right)\cdot \alpha_{\omega -h}\\ {BB}_{h}=\left(1-\alpha_{u-h}\right)\cdot \left(1-\Gamma_{nl-h}\right)\cdot \alpha_{n-h}\cdot \left(1-\Gamma_{wl-h}\right)\cdot \alpha_{\omega -h}+\left(1-\alpha_{n-h}\right)\cdot \\ \left(1-\Gamma_{wl-h}\right)\cdot \alpha_{\omega -h}+\left(1-\alpha_{n-h}\right)\cdot \Gamma_{nr-h}\cdot \alpha_{n-h}\cdot \left(1-\Gamma_{wl-h}\right)\cdot \alpha_{\omega -h}\\ {dd}_{h}=\left(1-\alpha_{w-h}\right)+\left(1-\alpha_{w-h}\right)\cdot \Gamma_{wr-h}\cdot \alpha_{\omega -h} \end{array}\right.$$

For the matched load branch, the matched load is directly connected to the switch through the waveguide port. Analyzing the brightness temperature $T_{L}^{\prime }$ after the isolator following the receiver switch, it consists of 3 components: the direct output of the matched load’s radiometric brightness temperature after attenuation by the switch; the contribution from the switch’s radiometric brightness temperature transmitted backward. The analysis yields

(9) $$T_{L}^{\prime }=T_{\omega -L}\left(\left(1-\Gamma_{sl-A}\right)\right)$$

Thus, the RF front-end transmission models for each branch of the radiometer have been established, yielding the relationship between the input brightness temperature of each branch and the brightness temperature received by the receiver. The calibration parameters used in this paper are shown in Table A2 and Table A3.

According to the variable relationships in Figure 10, it can be seen that

(10) $${T^{\prime }}_{h}\cdot t_{2}+{T^{\prime }}_{A}\cdot t_{1}={T^{\prime }}_{L}\cdot (t_{1}+t_{2})$$

From the above equation, it can be derived that

(11) $${T^{\prime }}_{A}=\frac{{T^{\prime }}_{L}\cdot \left(t_{1}+t_{2}\right)-{T^{\prime }}_{h}\cdot t_{2}}{t_{1}}$$

Then $T_{A}$ can be solved.

(12) $$T_{A}=\frac{T_{A}^{\prime }/\left(1-\Gamma_{sl-A}\right)-{bb}_{A}\cdot T_{\omega -A}}{{aa}_{A}}$$

### 3.2. Radiometer Calibration Experiment

**Figure 16:**
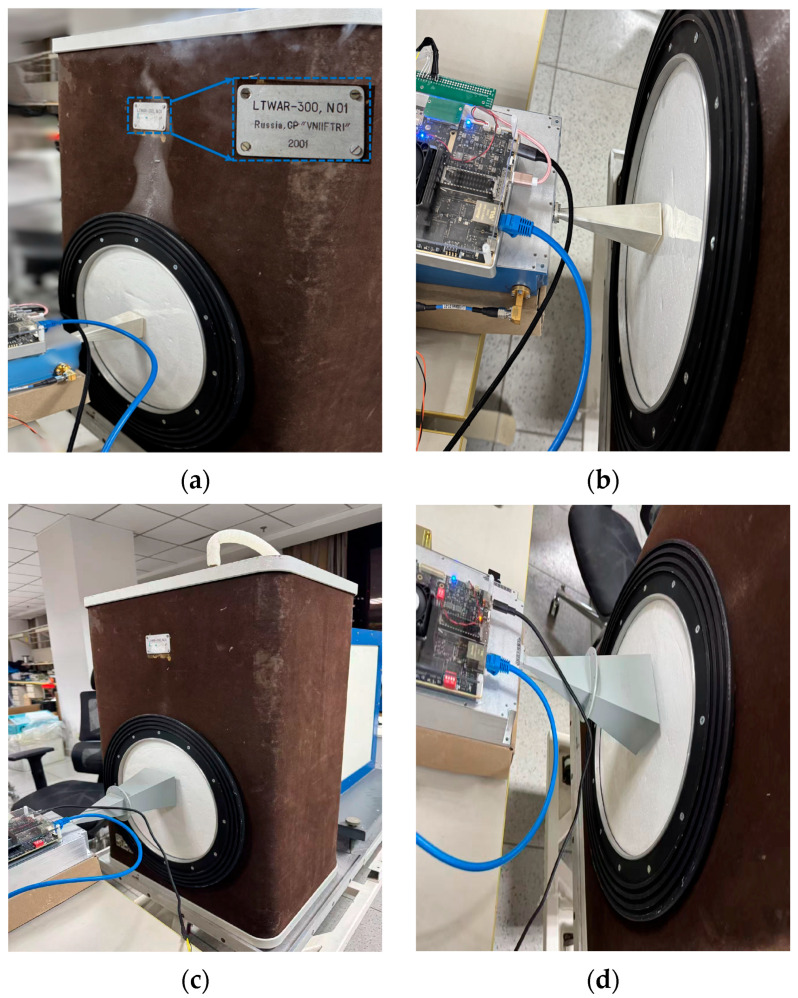
Cold source calibration field photo. (**a**) V-band overall image; (**b**) V-band local image; (**c**) K-band overall image; (**d**) K-band local image.

This system employs liquid nitrogen as a cold calibration source to calibrate the hot source. Although the temperature stabilization measures for the hot source are robust, they cannot be regarded as perfectly matched; therefore, a cold source is required as a reference for calibration. The experiment was conducted in a laboratory environment with a temperature of 25.6 °C, atmospheric pressure of 1011.5 hPa, and relative humidity of 15%. The laboratory temperature remained stable throughout the measurement period, with no external electromagnetic interference observed. Liquid nitrogen was poured into the LTWAR-300 cold source calibration chamber, and the aperture temperature of the cold source was 80.3 K. The antenna aperture was aligned facing the cold source, as shown in Figure 16, while the hot source port was connected to the hot source module. The calibration process using liquid nitrogen lasted for 12 h. During this period, the system was shut down, allowed to cool, and then restarted to perform repeated calibrations, with a total of 5 repetitions.

Here, $T_{A}$ is considered a known temperature, which is 80.3 K. The value of ${T^{\prime }}_{A}$ is calculated through Equation (11), and then ${T^{\prime }}_{h}$ is obtained.

(13) $${T^{\prime }}_{h}=\frac{{T^{\prime }}_{L}\cdot \left(t_{1}+t_{2}\right)-{T^{\prime }}_{A}\cdot t_{1}}{t_{2}}$$

The actual injection temperature of the hot source $T_{h}$ can be calculated through Equation (13).

(14) $$T_{h}=\frac{\frac{T_{h}^{\prime }}{\left(1-\Gamma_{sl-A}\right)}-{BB}_{h}\cdot T_{u-h}+{dd}_{h}\cdot T_{\omega -h}}{{aa}_{h}}$$

After calibration of the hot source, the cold source was measured again to verify the calibration results. Additionally, a blackbody was used for further verification.

**Figure 17:**
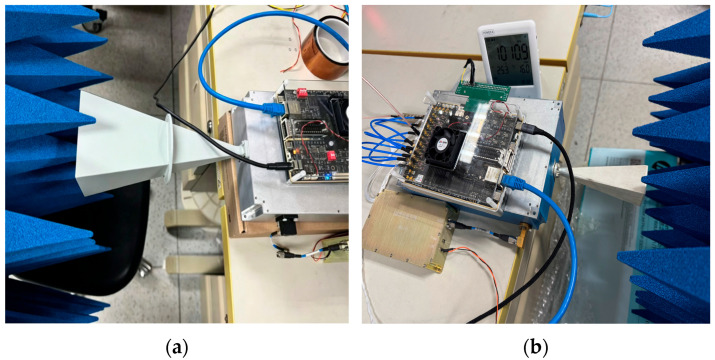
Blackbody verification of calibration results, field photo. (**a**) K-band; (**b**) V-band.

The blackbody experiment was conducted in a laboratory with a temperature of 25.3 °C, atmospheric pressure of 1010.9 hPa, and humidity of 16%. The blackbody utilizes pyramid-shaped microwave-absorbing material, processed into a four-sided pyramid array structure from carbon-containing polyurethane foam. The antenna aperture was aligned with the blackbody, as shown in Figure 17, with the hot source port connected to the hot source module.

## 4. Result

### 4.1. Calibration Result

**Figure 18:**
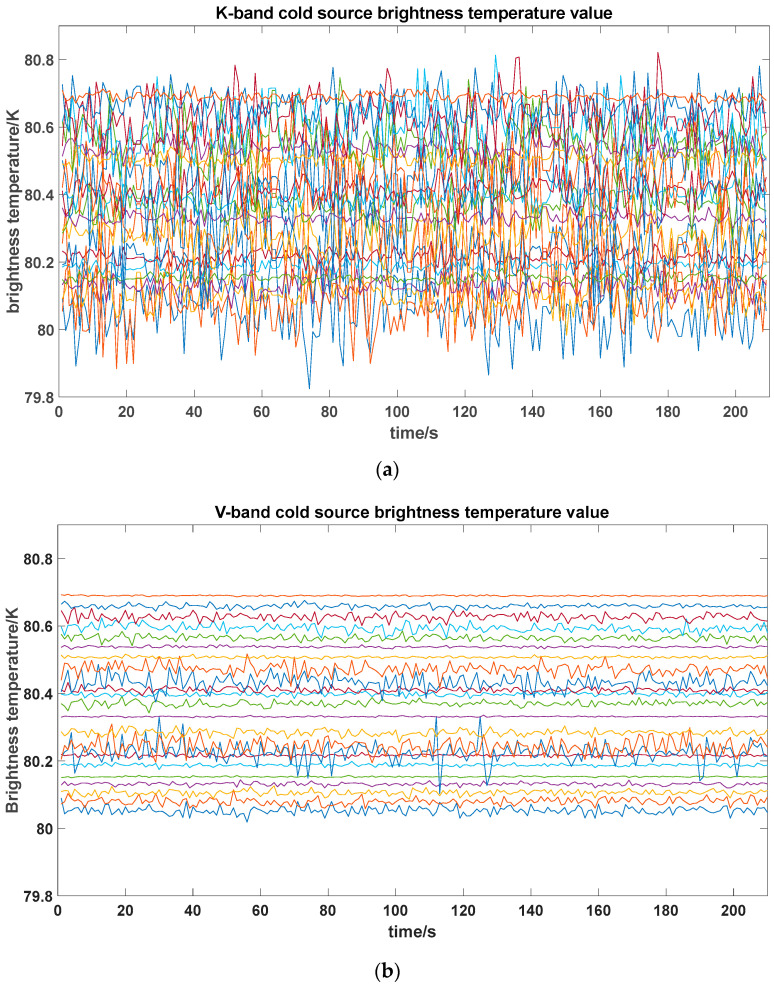
Cold source calibration result photo. (**a**) K-band; (**b**) V-band.

**Figure 19:**
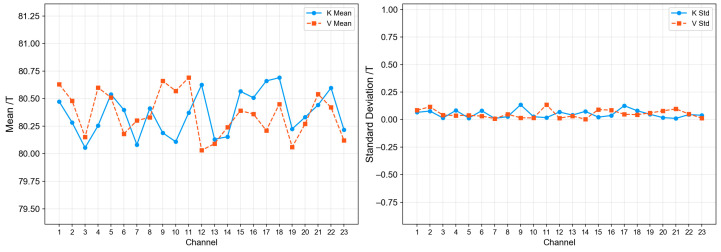
Cold source measurement result analysis.

After the cold source calculation, the actual hot source temperature $T_{h}=341.85K$. The calibrated system was used to measure the cold source again. The brightness temperature values of the antennas for each channel are shown in Figure 18, and the mean values and standard deviations of the brightness temperatures for each channel are shown in Figure 19. From the figure, the brightness temperatures of the 23 channels in both K-band and V-band fluctuate around 80 K, with a relatively concentrated overall distribution and no obvious drift or abnormal jump phenomena.

From the statistical results, the consistency among channels is good, with mean values fluctuating within the range of 80.05~80.69 K. Specifically, the K-band mean ranges from 80.053~80.690 K, and the V-band mean ranges from 80.03~80.69 K, with mean deviations within 0.7 K for both bands. The K-band standard deviation is 0.011~0.13 K, and the V-band standard deviation is 0.003~0.13 K, with most channels having standard deviations less than 0.09 K.

**Figure 20:**
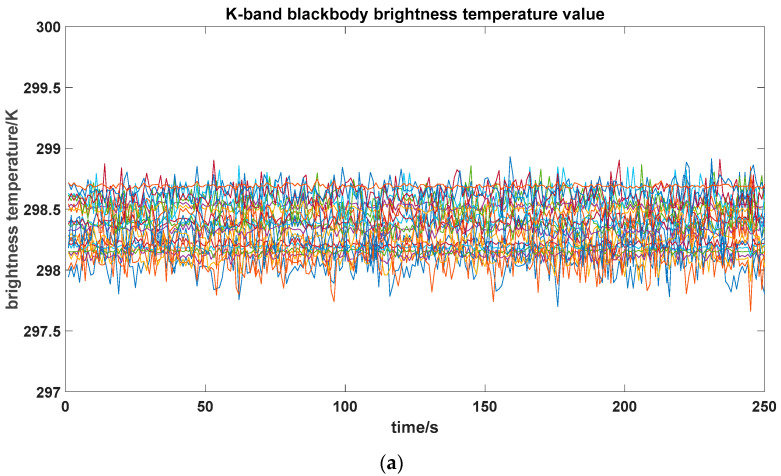

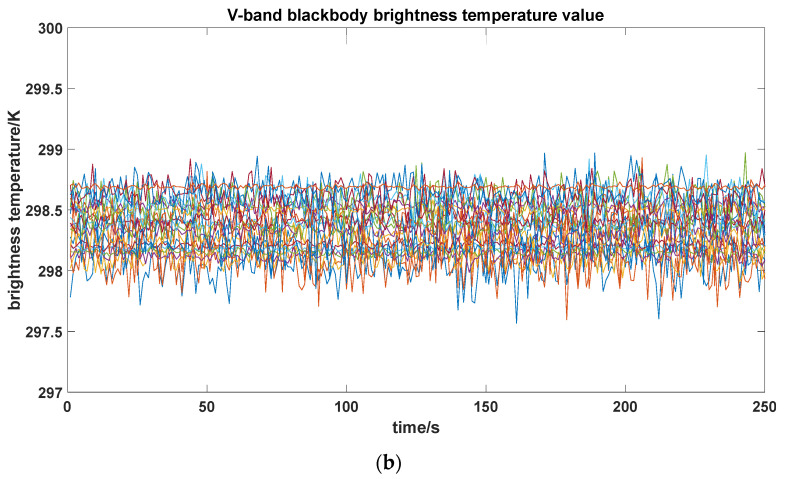
Blackbody calibration verification result photo. (**a**) K-band; (**b**) V-band.

**Figure 21:**
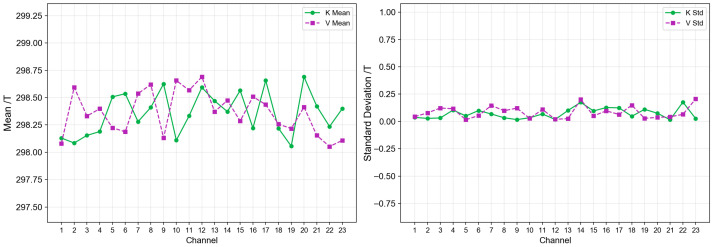
Blackbody calibration verification result analysis.

In the blackbody verification experiment, the brightness temperature values of the antennas for each channel are shown in Figure 20, and the mean values and standard deviations of the brightness temperatures for each channel are shown in Figure 21. From the figure, it can be seen that the brightness temperatures of the 23 channels in both K-band and V-band fluctuate around 298 K, with a relatively concentrated overall distribution and no obvious drift or abnormal jump phenomena.

From the statistical results, the consistency among channels is good, with mean values fluctuating within the range of 298.05~298.69 K. Specifically, the K-band mean ranges from 298.055 K, and the V-band mean ranges from 298.69 K, with mean deviations within 1 K for both bands. The K-band standard deviation is 0.15 K, and the V-band standard deviation is 0.21 K. The stability and consistency of all channels meet the accuracy requirements for radiometer brightness temperature measurements.

### 4.2. Comparison with Existing Systems

Table 5 compares the specifications of the developed Dicke radiometer with several state-of-the-art ground-based microwave radiometers. The BHU-K80 adopts a total-power architecture, achieving a sensitivity of 0.1–0.2 K with 2–6 s integration time. The MP-3000A delivers a sensitivity of 0.1–1.0 K with 0.01–2.5 s integration time. The RPG-HATPRO-G5 provides seven channels in each of the K- and V-bands, with sensitivities of 0.10 K (K-band) and 0.20 K (V-band) at 1 s integration.

The radiometer developed in this work features 23 channels in each of the K- and V-bands, achieving measured sensitivities of 0.15 K and 0.21 K, respectively, at 1 s integration, which are comparable to those of the RPG-HATPRO-G5. Despite significantly more channels than both the MP-3000A and the RPG-HATPRO-G5, the sensitivity remains competitive, validating the effectiveness of the proposed channelized receiver design.

## 5. Discussion

The digitization significantly reduces the complexity of the radiometer system while effectively ensuring channel consistency and system linearity. The error correction capability in the digital domain substantially improves system performance, which is fully validated by the experimental results.

The SDR-based digital architecture transforms the stringent integration time requirements in traditional modulation and demodulation processes into flexible digital implementations. By introducing an adaptive integration method, digital zero-balancing is achieved, making the system architecture more compatible with digital Dicke radiometer designs.

The front-end design employs RF switches to replace directional couplers, addressing the poor out-of-band suppression performance of couplers and significantly improving impedance matching characteristics. This system is implemented using a combination of two single-pole double-throw switches, though a single-pole triple-throw switch can also be adopted. The core requirement is to ensure that the total integration time for the antenna and hot source equals the integration time for the matched load.

The application of SDR technology substantially simplifies the system structure. Low-frequency radiometers can directly perform RF sampling, while high-frequency systems only require frequency conversion into the SDR sampling bandwidth to achieve digital reception. This architecture exhibits excellent scalability and can be conveniently extended to radiometer systems operating at other frequency bands.

## 6. Conclusions

The SDR-based digital architecture effectively simplifies the system structure, achieves high consistency among multiple channels, and significantly improves the nonlinearity of detector devices. For wideband, multi-channel systems, the SDR solution can significantly enhance processing efficiency, making it particularly suitable for applications such as water vapor and oxygen absorption bands. For higher frequency bands (e.g., 183 GHz, 115 GHz, etc.), digital reception can be achieved by simply frequency-converting the signal into the SDR sampling bandwidth.

The digital Dicke radiometer reduces system complexity and offers greater flexibility. The integration time can be flexibly adjusted digitally and configured according to actual observation requirements, demonstrating excellent applicability.

The development and experimental results of this paper show that the system exhibits good consistency across all channels. In cold-source and blackbody verification experiments, the mean deviations of K-band and V-band are within 0.7 K and 1 K, respectively, with standard deviations better than 0.13 K and 0.21 K, and most channels better than 0.09 K and 0.15 K. The calculated sensitivity at 1 s integration time is 0.06 K, and the measured sensitivities are 0.15 K (K-band) and 0.21 K (V-band), which are comparable to those of the RPG-HATPRO-G5.

After subsequent environmental hardening, this prototype can be applied in the field of atmospheric remote sensing. By measuring atmospheric brightness temperature data and combining it with radiosonde observation data to construct a dataset, a neural network model can be trained to retrieve atmospheric temperature and humidity profiles.

## Figures and Tables

**Table 1:** Key performance parameters of the radiometer.

Parameter	K-Band
Frequency	K: 21.3~31.7 GHz; V: 50.8~61.2 GHz
Channels	K: 23; V: 23
Bandwidth	288 MHz (1~4, 19~23); 400 MHz (5~18)
Measurement range	0~400 K
Sensitivity	K: 0.15 K; V: 0.21 K
Consistency	≤1 K
Hot source	341.85 K
Inertial	30 ms
Weight	18 kg
Dimensions	30 × 20 × 65 cm

**Table 2:** Antenna parameter table.

Parameter	K-Band	V-Band
3 dB beamwidth	5~12°	5–7°
dimensions	95 × 80 × 267.5 mm	40 × 33 × 123 mm
waveguide designation	BJ260	BJ620
gain	25 dBi	25 dBi
VSWR	1.25	1.25

**Table 3:** ADC sampling rate and clock configuration.

Signal	Analog Signal Frequency	Sampling Rate	ADC Clock	Nyquist Zone	Digital Signal Frequency
$I_{1},Q_{1}$	DC~2.2 GHz	5.0 GHz	500 MHz	1	DC~2.2 GHz
$I_{2},Q_{2}$	2.3~3.7 GHz	4.0 GHz	500 MHz	2	1.7~0.3 GHz
$I_{3},Q_{3}$	3.8~5.2 GHz	3.6 GHz	360 MHz	3	0.2~1.6 GHz

**Table 4:** Prototype filter characteristics table.

Prototype Filter	Analog Signal Frequency	Sampling Rate	Stopband Attenuation	Ripple	Rectangular Ratio	Coefficient Number
${Filter}_{I_{1}\_Q_{1}}$	200 MHz	250 MHz	70 dB	0.5 dB	0.8	250
${Filter}_{I_{2}\_Q_{2}}$	200 MHz	250 MHz	70 dB	0.5 dB	0.8	200
${Filter}_{I_{3}\_Q_{3}}$	180 MHz	144 MHz	70 dB	0.5 dB	0.8	250

**Table 5:** Comparison of ground-based microwave radiometer specifications.

Parameter	Specifications			
Name	BHU-K80 [10]	MP-3000A [10]	RPG-HATPRO-G5 [10]	THIS WORK
Frequency	22~31 GHz	22~30 GHz 51~59 GHz	22.24~31.40 GHz 51.26~58.00 GHz	22.3~31.7 GHz 50.8~61.2 GHz
Channels	80	21 + 14	7 + 7	23 + 23
Bandwidth	100 MHz	300 MHz	230 MHz (11 channels); 600 MHz (56.66 GHz); 1000 MHz (57.30 GHz); 2000 MHz (58.00 GHz)	288 MHz (1~4,19~23) 400 MHz (5~18)
Sensitivity	0.1 K–0.2 K	0.1 K–1 K	0.10 K; 0.20 K	0.15 K; 0.21 K
Integration time	2–6 s	0.01–2.5 s	1 s	1 s

## Data Availability

The data that support the findings of this study are available from the corresponding author upon reasonable request.

## Appendix A

**Table A1:** K-band (water vapor)/V-band (Oxygen) frequency allocation table.

Channel No.	K Center Frequency/GHz	V Center Frequency/GHz	Bandwidth/MHz
1	21.46	50.96	±144 M
2	21.82	51.32	±144 M
3	22.18	51.68	±144 M
4	22.54	52.04	±144 M
5	23.00	52.50	±200 M
6	23.50	53.00	±200 M
7	24.00	53.50	±200 M
8	24.50	54.00	±200 M
9	25.00	54.50	±200 M
10	25.50	55.00	±200 M
11	26.00	55.50	±200 M
12	26.50	56.00	±200 M
13	27.00	56.50	±200 M
14	27.50	57.00	±200 M
15	28.00	57.50	±200 M
16	28.50	58.00	±200 M
17	29.00	58.50	±200 M
18	29.50	59.00	±200 M
19	30.00	59.50	±200 M
20	30.46	59.96	±144 M
21	30.82	60.32	±144 M
22	31.18	60.68	±144 M
23	31.54	61.04	±144 M

**Table A2:** K-band calibration factor.

F (GHz)	${aa}_{A}$	${bb}_{A}$	${aa}_{h}$	${BB}_{h}$	${dd}_{h}$	${AA}_{L}$
21.46	0.614545305	0.370285698	0.430026165	0.090789685	0.399267677	1
21.82	0.583667191	0.401111499	0.494622477	0.104918945	0.373086576	1
22.18	0.569082721	0.418625725	0.504842927	0.108590768	0.369027333	1
22.54	0.574514189	0.417712155	0.496939077	0.108378217	0.370111525	1
23	0.616792149	0.381339767	0.516862608	0.11165708	0.341842329	1
23.5	0.631106181	0.362277791	0.560029484	0.117819723	0.308569246	1
24	0.602651453	0.380466362	0.558446735	0.117144599	0.315034753	1
24.5	0.580904744	0.407910407	0.49485544	0.105194447	0.364828916	1
25	0.568924354	0.430160219	0.484690875	0.104781356	0.383191666	1
25.5	0.55076981	0.451572134	0.479687936	0.105741437	0.390036698	1
26	0.541637597	0.4556988	0.402364124	0.090056877	0.433299993	1
26.5	0.541700663	0.421232963	0.328653729	0.074492768	0.442193581	1
27	0.539977483	0.433224227	0.381705629	0.085372013	0.452856297	1
27.5	0.545138939	0.444924183	0.439928116	0.095277947	0.426663464	1
28	0.569428239	0.429051774	0.4252934	0.089890772	0.432832111	1
28.5	0.619438756	0.382489959	0.370476135	0.076704186	0.455755917	1
29	0.63310891	0.361867678	0.45414355	0.093351505	0.405628034	1
29.5	0.589570442	0.3983655	0.51749019	0.106339504	0.373154621	1
30	0.543192002	0.441592502	0.48770269	0.099003294	0.411768008	1
30.46	0.560644803	0.42691077	0.479562184	0.095547118	0.420728615	1
30.82	0.624653153	0.365113162	0.491738261	0.096609949	0.388309311	1
31.18	0.705690675	0.276329128	0.523387498	0.102374572	0.325032462	1
31.54	0.720324442	0.226665204	0.505558154	0.09838997	0.295045918	1

**Table A3:** V-band calibration factor.

F (GHz)	${aa}_{A}$	${bb}_{A}$	${aa}_{h}$	${BB}_{h}$	${dd}_{h}$	${AA}_{L}$
50.96	0.542559898	0.457368251	0.322566391	0.149088701	0.520738772	1
51.32	0.542695038	0.457970459	0.315666064	0.144962181	0.530015639	1
51.68	0.548328128	0.452383475	0.315121143	0.14366377	0.529881621	1
52.04	0.552251703	0.447748888	0.307174583	0.138949155	0.53582891	1
52.5	0.531781166	0.46704323	0.279317264	0.124948539	0.567061705	1
53	0.557587943	0.441989328	0.291546926	0.128796604	0.558854001	1
53.5	0.537460304	0.462973262	0.264775080	0.115484842	0.599438842	1
54	0.531876611	0.463722006	0.200706852	0.089772546	0.65544157	1
54.5	0.525691740	0.468757457	0.166857004	0.076537466	0.689380242	1
55	0.595839995	0.399559448	0.217139493	0.102084823	0.619375776	1
55.5	0.592995435	0.403470958	0.275817365	0.130670151	0.56490874	1
56	0.633282250	0.363217089	0.327706777	0.156431393	0.507829117	1
56.5	0.577877028	0.419552524	0.294328117	0.141570003	0.550550682	1
57	0.562352399	0.437856688	0.233698980	0.109895663	0.601267613	1
57.5	0.532490250	0.469447176	0.181084967	0.083221451	0.666808519	1
58	0.552228292	0.449122539	0.185415531	0.083310846	0.675404337	1
58.5	0.578538269	0.423477962	0.214409162	0.094655421	0.653385631	1
59	0.573632785	0.427776899	0.219981065	0.095404424	0.646932067	1
59.5	0.580056122	0.416428698	0.208235849	0.088554849	0.645346041	1
59.96	0.596417089	0.394854653	0.206709692	0.087593208	0.633836535	1
60.32	0.593696314	0.397140072	0.223712499	0.094462404	0.615222139	1
60.68	0.599561300	0.389407359	0.263914438	0.110919468	0.572968084	1
61.04	0.530934387	0.445228595	0.281456909	0.118206995	0.561837906	1
